# Monocular Clinical Outcomes and Range of Near Vision following Cataract Surgery with Implantation of an Extended Depth of Focus Intraocular Lens

**DOI:** 10.1155/2018/8205824

**Published:** 2018-12-13

**Authors:** Rahul T. Pandit

**Affiliations:** ^1^Blanton Eye Institute, Houston Methodist Hospital, Houston, TX, USA; ^2^Weill Cornell Medicine, New York, NY, USA

## Abstract

**Importance:**

A full range of near and intermediate vision has not been clinically evaluated for the Symfony extended depth of focus intraocular lens (EDOF IOL).

**Background:**

To evaluate the monocular range of near visual acuity with an EDOF IOL.

**Design:**

Retrospective case series.

**Participants:**

Consecutive patients of a single surgeon from January 2017 through March 2018.

**Methods:**

Phacoemulsification with implantation of an EDOF IOL.

**Main Outcome Measures:**

Uncorrected distance visual acuity (UDVA), uncorrected near visual acuity (UNVA), corrected distance visual acuity (CDVA), distance-corrected near visual acuity (DCNVA), range of DCNVA, and optimal near focal length.

**Results:**

Seventy-six eyes of 48 patients (34 or 71% female, mean age: 68 years) were included with a mean follow-up of 68 days. Mean values were as follows: logarithm of the minimum angle of resolution (logMAR) UDVA 0.02 ± 0.09, logMAR UNVA 0.12 ± 0.09 at a mean distance of 51 cm, logMAR CDVA −0.05 ± 0.07, logMAR DCNVA 0.08 ± 0.07 at a mean distance of 51 cm, and a spherical equivalent of −0.16 diopters ±0.35. Percentage of eyes achieving DCNVA of 20/30 were 84% at 36 cm, 92% at 41 cm, 99% at 51 cm, 93% at 61 cm, and 74% at 71 cm. DCNVA of 20/40 or better was achieved in nearly 100% of eyes over a range of 35 cm.

**Conclusions and Relevance:**

The Symfony EDOF IOL achieved excellent distance visual acuity while providing a 35 cm range of near visual acuity at levels useful for many tasks in nearly all patients.

## 1. Introduction

Presbyopia can be a significant factor affecting quality of life in the developed and developing world [1, 2]. In 2014, an FDA panel developed standards for a new category of presbyopia-correcting intraocular lenses (IOLs) [3]. Deemed “extended depth of focus (EDOF)” or “extended range of vision (EROV),” these IOLs are distinguished from traditional multifocal and trifocal lenses in their more elongated defocus curve peaks without the traditional bimodal or trimodal peaks seen with multifocal or trifocal intraocular lenses [4]. This presumably translates to a more continuous range of near vision instead of multiple points of focus.

**Figure 1:**
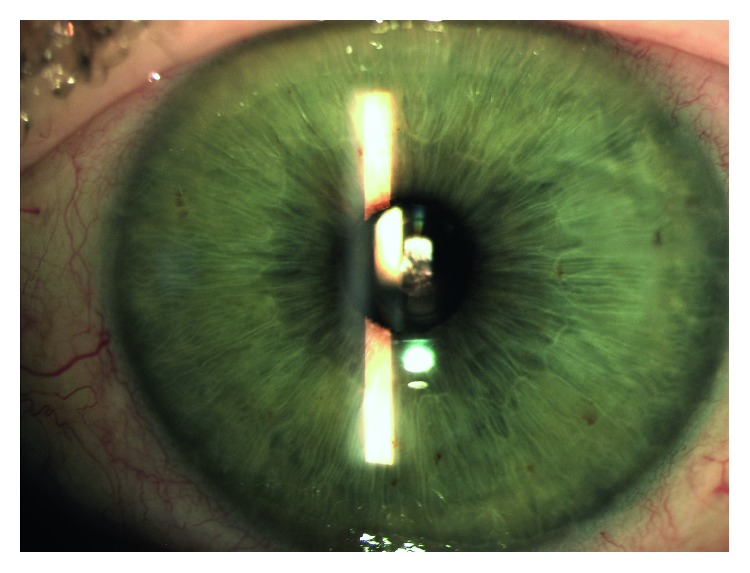
Slit lamp photo of Symfony EDOF IOL implanted in a study eye.

Currently, the only EDOF IOL available in the United States is the Tecnis Symfony extended depth of focus IOL (Johnson & Johnson Vision, NJ) [5, 6], which is available in a standard version as well as toric versions. This lens uses diffractive optics to enhance quality of vision by increasing depth of focus and actively correcting for spherical aberration (Figure 1) [7]. The Symfony IOL has been shown to provide enhanced near vision outcomes as compared to monofocal lenses and has been compared to a variety of presbyopia-correcting technologies [8–21]. Published studies have mostly reported clinical outcomes from bilateral implantation of this lens in patients, typically limiting their endpoints to two or a maximum of three near points of focus [11, 14, 15, 17, 22]. In some cases, the lens has been evaluated binocularly with planned mini-monovision [19] or with another presbyopia-correcting lens in the contralateral eye [23]. No prior study has evaluated multiple near focal distances to establish a monocular range of near visual acuity in eyes with the Symfony EDOF IOL. This study was designed to evaluate monocular outcomes of the range of near vision in eyes implanted with the Symfony IOL by a single surgeon in a routine clinical setting and to establish the average preferred monocular near point of focus with this lens.

## 2. Patients and Methods

A retrospective chart review was conducted for all patients undergoing phacoemulsification by a single surgeon (RTP) with implantation of a Symfony IOL (ZXR00) or Symfony toric IOL (ZXT150, ZXT225, ZXT300, and ZXT375; Johnson & Johnson Vision, NJ) from January 2017 through March 2018. Exclusion criteria included lack of follow-up beyond 21 days postoperatively, concurrent ocular conditions limiting best corrected distance visual acuity to less than or equal to 20/40 Snellen, visually significant posterior capsular opacification (PCO) requiring YAG laser capsulotomy within 4 months of phacoemulsification, or visually significant dysphotopsia requiring IOL exchange. Patients with prior corneal refractive surgery including radial keratotomy were not excluded. An institutional review board approval was obtained by the Houston Methodist Research Institute, Houston, TX.

Phacoemulsification was performed using a standard microinvasive cataract surgery technique [24]. Both manual as well as femtosecond laser-assisted cataract surgery (FLACS) cases were included.

Visual acuity was reported at the first postoperative visit after phacoemulsification when eyes were considered stable (no concurrent postoperative ocular conditions affecting DCVA including unresolved iritis, cystoid macular edema, and keratitis sicca). Near visual acuity was measured using a card on a phoropter rod with the patient's distance refraction placed in the phoropter.

Primary endpoints were uncorrected distance visual acuity (UDVA), uncorrected near visual acuity (UNVA), corrected distance visual acuity (CDVA), distance-corrected near visual acuity (DCNVA), range of DCNVA measured at 36, 41, 51, 61, and 71 cm, UNVA optimal focal length, and DCNVA optimal focal length. These distances were based on the following factors: approximate reading focal lengths seen with traditional multifocal lenses in the same IOL platform used by the surgeon (available in add powers of +4.00, +3.25, and +2.75 diopters at the IOL plane), average focal distance to desktop computer screens, and the maximal length of the phoropter near vision rod (28 inches or 71 cm). Secondary endpoints included incidence of posterior capsular opacification requiring YAG laser capsulotomy and incidence of lens exchange due to patient dissatisfaction.

### 2.1. Statistical Analysis

Data analysis was performed using Microsoft Excel 2016 (Microsoft Inc., WA). Snellen visual acuities were converted to logarithm of the minimal angle of resolution (logMAR) for calculation of mean and standard deviation values.

## 3. Results

**Figure 2:**
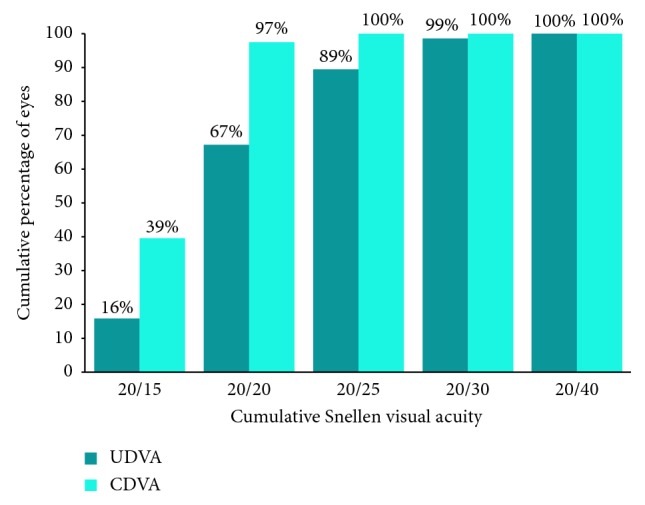
Cumulative monocular uncorrected and corrected distance visual acuity outcomes.

**Figure 3:**
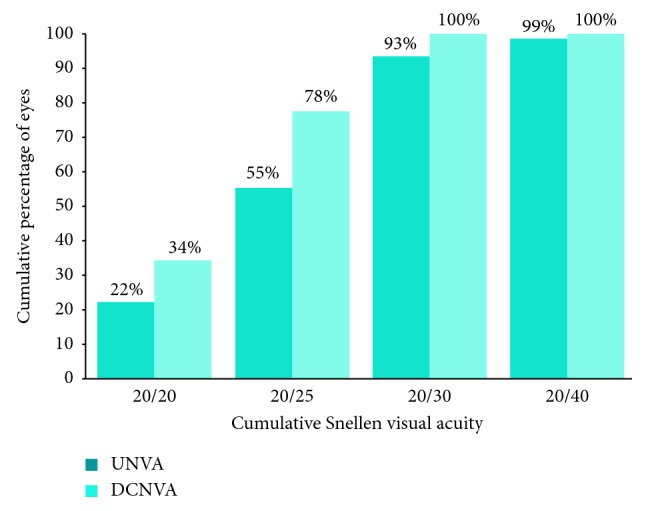
Cumulative monocular uncorrected and corrected near visual acuity outcomes.

A total of 100 eyes of 65 patients were identified that underwent phacoemulsification during the study period with implantation of a Symfony EDOF IOL. Twenty-four eyes of 18 patients were excluded based on the following criteria: lack of adequate follow-up (7 eyes), concurrent ocular conditions limiting DCVA to less than or equal to 20/40 (10 eyes), postoperative PCO requiring YAG capsulotomy (6 eyes), and intractable dysphotopsia requiring IOL exchange (1 eye). The remaining 76 eyes of 48 patients (34 or 71% female) met inclusion criteria. Sixty-nine (91%) of these eyes underwent FLACS, and 7 (9%) underwent concurrent trabectome surgery along with phacoemulsification. Sixty eyes (79%) were implanted with the Symfony IOL, and the remaining 16 eyes with the Symfony toric IOL. The mean age of patients was 68 years (standard deviation 7.3; range 45–82), and the mean follow-up was 68 days (standard deviation 38; range 27–167). Mean values for UDVA, UNVA, CDVA, DCNVA, spherical equivalent, and optimal focal distance for near vision are reported in Table 1. Cumulative distance visual acuity outcomes and near visual acuity outcomes are outlined in Figures 2 and 3, respectively.

**Figure 4:**
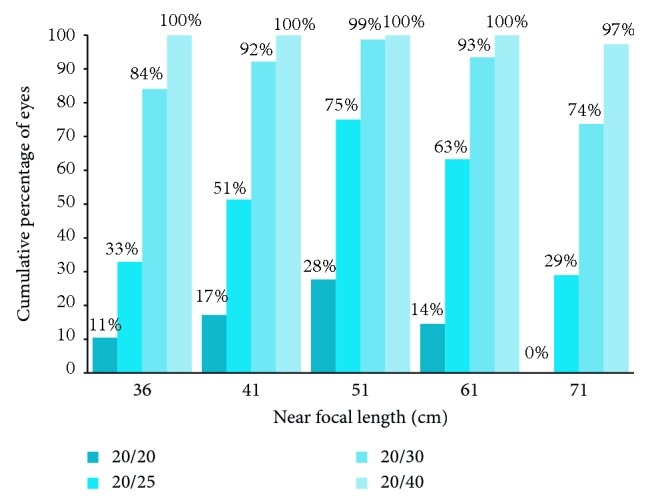
Cumulative near visual acuity outcomes at various levels of Snellen acuity from 36 to 71 centimeters (cm).

Percentage of eyes achieving DCNVA of greater than or equal to 20/20, 20/25, 20/30, or 20/40 at the various near focal lengths tested from 36 to 71 cm are displayed in Figure 4.

**Figure 5:**
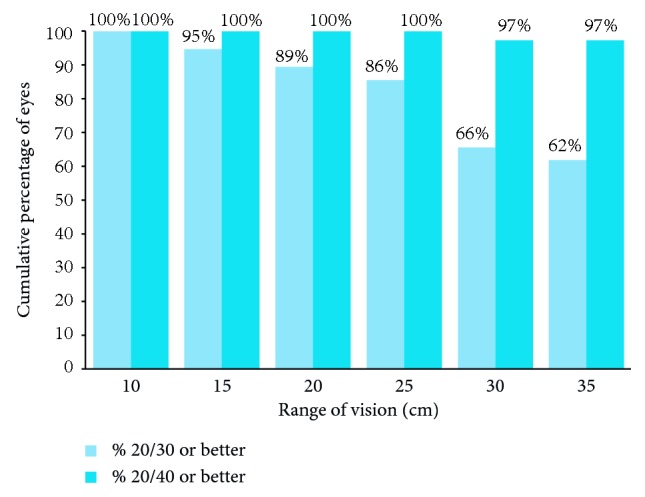
Range in centimeters (cm) of near visual acuity at 20/30 or better and 20/40 or better.

The range of DCNVA at levels of 20/30 or better and 20/40 or better is displayed in Figure 5. Overall, 97% of eyes achieved 20/40 or better DCNVA over a range of 35 cm, and all eyes exhibited 20/40 or better DCNVA over a range of 25 cm (Figure 5).

## 4. Discussion

The Symfony extended depth of focus intraocular lens, like many multifocal intraocular lenses, relies on diffractive optics to focus light at different distances from the lens. Benchmark testing of this lens suggests that it can achieve a high level of visual acuity over a range of vision as opposed to providing a distinct near point of focus as with a traditional multifocal lens [7]. To achieve an adequate level of visual functioning over a range of vision, the optics of the Symfony IOL correct for different types of aberration, including chromatic and spherical, providing an extremely high peak in its defocus curve, which then using diffractive steps can result in a highly functional range of vision over a normal acuity range. The clinical trials used for FDA approval for this lens reported results of monocular and binocular acuity at 40 and 66 cm, in addition to distance. Intermediate and near visual acuity outcomes at 20/40 and better were reported in these clinical trials. Other studies have evaluated two and up to three near focal lengths, typically binocularly [11, 14, 15, 17]. This study included a total of five near points of focus to fully evaluate the range of vision afforded by this lens.

We chose to evaluate monocular visual acuity results only, as we felt that the effect of binocular summation of visual input might lead to falsely elevated visual acuity outcomes. Furthermore, many of our patients are only eligible for implantation of this IOL in one eye since their contralateral eye does not have a visually significant cataract.

This study demonstrated that patients achieved an excellent level of DCNVA at intermediate ranges of near vision. Optimal near focal point was at a range most compatible with computer distance and arm's length placement of near reading material, consistent with the clinical trial results of this IOL. Furthermore, this study demonstrated that patients achieved a satisfactory level of distance-corrected near visual acuity over an extended range of near vision, as close as 36 cm and as far as 71 cm in some eyes. At 36 and 71 cm, 34% and 30% of eyes, respectively, had a visual acuity of 20/25 or better. All patients demonstrated 25 cm of range of near vision at 20/40 or better, and 97% of eyes had the same level of acuity over the 35 cm range we tested. The FDA clinical trial results measured near and intermediate visual acuity up to the 20/40 level, beyond that, eyes were grouped in ranges of vision. We feel that 20/40 on a near card represents a size of print that most closely resembles daily reading tasks such as computer and tablet screen print, smartphones, and many magazines and books.

This data set included some eyes with ocular comorbidities traditionally considered as relative contraindications for implantation with a MFIOL. We took this approach based on clinical understanding of the optics of this lens resulting in less diffractive loss of light than with traditional MFIOLs, early outcomes in patients with such comorbidities who underwent EDOF IOL implantation, and a thorough informed consent process explaining the risks, benefits, and alternatives to phacoemulsification with implantation of an EDOF IOL. These eyes are included in the data analysis. One patient had moderate primary open-angle glaucoma and a prior Crystalens IOL implanted elsewhere in the contralateral eye; as a result of her dissatisfaction with the contralateral eye and a strong desire for an extended range of vision, she requested an EDOF in her operative eye. Several eyes had prior myopic laser keratorefractive surgery, two of which had significant irregular astigmatism and higher order aberrations on corneal dual Scheimpflug with placido imaging. Limited study data are available on the results of EDOF implantation in postrefractive eyes [25–27], but our experience with multifocal IOLs in such cases led us to proceed. One eye had prior vitrectomy for a macular hole with persistent distortion in the vision despite anatomic closure of the hole; she was able to achieve excellent acuity despite persistent microdistortion after phacoemulsification surgery. One eye had preexisting 16-cut radial keratotomy (RK) with satisfactory outcomes.

Near vision was reported looking at a near card for acuity measurements in ideal lighting conditions, and the range of vision was estimated based on visual acuity measurements at specific numeric distances on the near phoropter rod. This methodology may have affected the study outcomes in two manners. It is possible that the range of DCNVA results might underestimate the true range of vision since measured distances were in steps of 10 cm (4 inches) and stopped at a distance of 71 cm (28 inches). On the other hand, the use of a near acuity card in ideal lighting conditions may overestimate real-world outcomes in patients who are reading text for an extended period, especially if surrounded by poor lighting. This of course would be mitigated in real-world situations through binocular summation in bilaterally implanted patients, which again was not the purpose of this study.

Twenty-four percent of eyes during the study period were excluded due to the aforementioned reasons. One of those patients, who had a past ocular history of myopic LASIK, suffered severely from a dysphotopsia at day and night reported since her first postoperative day exam. This dysphotopsia was not described as “bright” or as “dark”, just an “edge of something like a contact lens” appearing constantly in her vision. Despite multiple discussions over multiple visits, the exact nature of her dysphotopsia was still unclear. As a result, her EDOF IOL was exchanged for a three-piece, monofocal, aspheric, silicone IOL placed in the capsular bag with reverse optic capture, which resolved her symptom completely.

Of the remaining excluded eyes, seven were lost to follow-up due to lack of geographic proximity to the surgeon. Though follow-up data could not be obtained on these patients, they were very satisfied in their early postoperative period in our clinic. Ten eyes were excluded due to concurrent ocular conditions. Implantation of the EDOF IOL in these patients was planned only after careful and thorough discussion about the potential risks and potential lack of positive near vision outcomes in these patients' eyes. Concurrent conditions included Fuchs corneal dystrophy requiring endothelial keratoplasty, epiretinal membrane, optic atrophy, and high ametropic amblyopia. An additional six eyes were excluded due to early significant posterior capsular opacification affecting visual acuity and requiring YAG laser capsulotomy within four months of their surgery. This represented 6% of the original cohort of patients, all of whom underwent FLACS, as did most of the study patients, and an incidence consistent with other studies of early PCO development [28].

This study was not designed to capture side effects or overall satisfaction rates of patients undergoing phacoemulsification with an EDOF IOL since detailed questionnaires were not administered asking about such symptoms as glare and haloes. It can only be stated that one patient suffered a significant dysphotopsia of uncertain etiology (lens design versus material versus capsule-IOL overlap) requiring a lens exchange. Nevertheless, a review of all clinic notes revealed that no patient meeting inclusion criteria was dissatisfied, and though many exhibited early dysphotopsias such as glare, only two patients spontaneously reported persistent moderate symptoms on their final postoperative visit during the study period. The first patient had a history of prior myopic photorefractive keratectomy (PRK) and experienced haloes. The second patient perceived glare indoors and outdoors, which were improving. None of these patients were prevented from driving at night due to their dysphotopsias. On the contrary, an interesting observation was that one study patient had undergone a monofocal lens implant with her cataract surgery in the contralateral eye two weeks prior to the study eye, and due to expected but unsatisfactory lack of near vision, regretted her decision and therefore opted for the EDOF lens in her second eye. She functioned well without glasses for many tasks but did require reading glasses for extended reading and small print.

This study is limited by its retrospective nature and the lack of a control group. However, all surgeries were performed by one surgeon and techniques for measuring visual outcomes in the clinic were standardized between visits and between technicians. We therefore feel that the retrospective design does not significantly limit the strength of the visual outcomes reported here. These results are relevant to demonstrating clinical outcomes of phacoemulsification with an EDOF IOL, especially since this data set included eyes that many would consider not ideal candidates for MFIOL implantation due to past ocular history.

## 5. Conclusion

This study demonstrates that the Symfony EDOF IOL achieves an excellent level of distance visual acuity while providing 35 cm of range of near visual acuity at a level useful for a variety of near visual tasks in nearly all patients.

## Figures and Tables

**Table 1:** Mean outcomes.

	Mean ± SD logMAR	Snellen equivalent
UDVA	0.02 ± 0.09	20/21
UNVA	0.12 ± 0.09	20/26
UNVA optimal distance, cm (in)	51 (20) ± 7	
CDVA	−0.05 ± 0.06	20/18
DCNVA	0.08 ± 0.07	20/24
DCNVA optimal distance, cm (in)	50 (20) ± 5	
Spherical equivalent, diopters	−0.16 ± 0.35	

UDVA = uncorrected distance visual acuity; UNVA = uncorrected near visual acuity; CDVA = corrected distance visual acuity; DCNVA = distance-corrected near visual acuity; cm = centimeters; in = inches.

## Data Availability

The Excel spreadsheet including data and graphs used to support the findings of this study is available from the corresponding author upon request.
